# Lipidomic signature of stroke recurrence after transient ischemic attack

**DOI:** 10.1038/s41598-023-40838-7

**Published:** 2023-08-22

**Authors:** F. Purroy, A. Ois, M. Jove, G. Arque, J. Sol, G. Mauri-Capdevila, A. Rodriguez-Campello, R. Pamplona, M. Portero, J. Roquer

**Affiliations:** 1 Clinical Neurosciences Group, Institut de Recerca Biomèdica de Lleida, UdL, Lleida, Spain; 2 Stroke Unit, Department of Neurology, Universitat de Lleida, Hospital Universitari Arnau de Vilanova, Avda Rovira Roure 80, 25198 Lleida, Spain; 3 Department of Neurology, Neurology Neurovascular Research Unit Hospital del Mar Research Institute (IMIM), Barcelona, Spain; 4 Experimental Medicine Department, Lleida University-Lleida Biomedical Research Institute (UdL-IRBLleida), 25198 Lleida, Spain; 5 Institut Català de la Salut (ICS), Atenció Primària, Lleida, Spain; 6 Research Support Unit Lleida, Fundació Institut Universitari per a la recerca a l’Atenció Primària de Salut Jordi Gol i Gurina (IDIAPJGol), Lleida, Spain

**Keywords:** Neuroscience, Diseases, Medical research, Neurology, Risk factors

## Abstract

While TIA patients have transient symptoms, they should not be underestimated, as they could have an underlying pathology that may lead to a subsequent stroke: stroke recurrence (SR). Previously, it has been described the involvement of lipids in different vascular diseases. The aim of the current study was to perform a lipidomic analysis to identify differences in the lipidomic profile between patients with SR and patients without. Untargeted lipidomic analysis was performed in plasma samples of 460 consecutive TIA patients recruited < 24 h after the onset of symptoms. 37 (8%) patients suffered SR at 90 days. Lipidomic profiling disclosed 7 lipid species differentially expressed between groups: 5 triacylglycerides (TG), 1 diacylglyceride (DG), and 1 alkenyl-PE (plasmalogen) [specifically, TG(56:1), TG(63:0), TG(58:2), TG(50:5), TG(53:7, DG(38:5)) and PE(P-18:0/18:2)]. 6 of these 7 lipid species belonged to the glycerolipid family and a plasmalogen, pointing to bioenergetics pathways, as well as oxidative stress response. In this context, it was proposed the PE(P-18:0/18:2) as potential biomarker of SR condition.

The observed changes in lipid patterns suggest pathophysiological mechanisms associated with lipid droplets metabolism and antioxidant protection that is translated to plasma level as consequence of a more intensive or high-risk ischemic condition related to SR.

## Introduction

Stroke is an important cause of disability and death globally, resulting in more than 6 million deaths per year^1,2^. A transient ischemic attack (TIA) is a form of stroke characterized by transient episodes of neurological deficits due to brain ischemia^3^. Despite the temporary nature of their symptoms TIA patients are at a significant risk of suffering a definitive ischemic stroke with persistent symptoms (stroke recurrence [SR]) particularly during the first three months of follow-up^4,5^. Interestingly, the risk of SR in TIA patients is heterogenous with some individuals having a high risk while others have a lower risk^6^. It is known that patients with intracranial or extracranial stenosis^6–8^, cardioembolism^9^, diffusion weighted imaging abnormalities^7,9^ and patients with repeated events^10^ or motor weakness^4,9^ have a higher risk. Furthermore, these differences in SR can vary based on sex as well^6^. Simultaneously, there has been a long-standing interest in the development of biomarkers for a considerable period of time. Biomarkers could provide valuable prognostic information^11^. It is important to note that due to TIA being a prevalent condition^2^, patients could be attended in centers without expertise or lacking the necessary technology. Therefore, identifying patients solely based on a blood test can be of interest. In this line, previous studies of our team predicted a significant role in the determination of lipids and their metabolites^12^. We observed how specific lysophosphatidylcholines (LysoPC[16:0] and LysoPC[20:4]) were significantly associated with SR. Lipids are involved in cardiovasculars diseases and acute myocardial infarction, not only a result of the retention of LDL-cholesterol and other cholesterol-rich apolipoprotein B-containing lipoproteins within the arterial wall but also as oxidative damage targets and the adaptation of lipid metabolism to ischemic processes^13^. Lipidomics, a subfield of metabolomics, involves the identification and quantification of the lipidome in biological systems. Lipidomics provides specific insight into the pathophysiologic mechanisms underlying ischemic stroke, and it would be a new strategy to describe biomarkers^14^.

The aim of the current study was to perform a lipidomic analysis among consecutive TIA patients to find differences in the lipidomic profile in plasma samples between patients with SR after 90 days and patients without.

## Results

**Figure 1:**
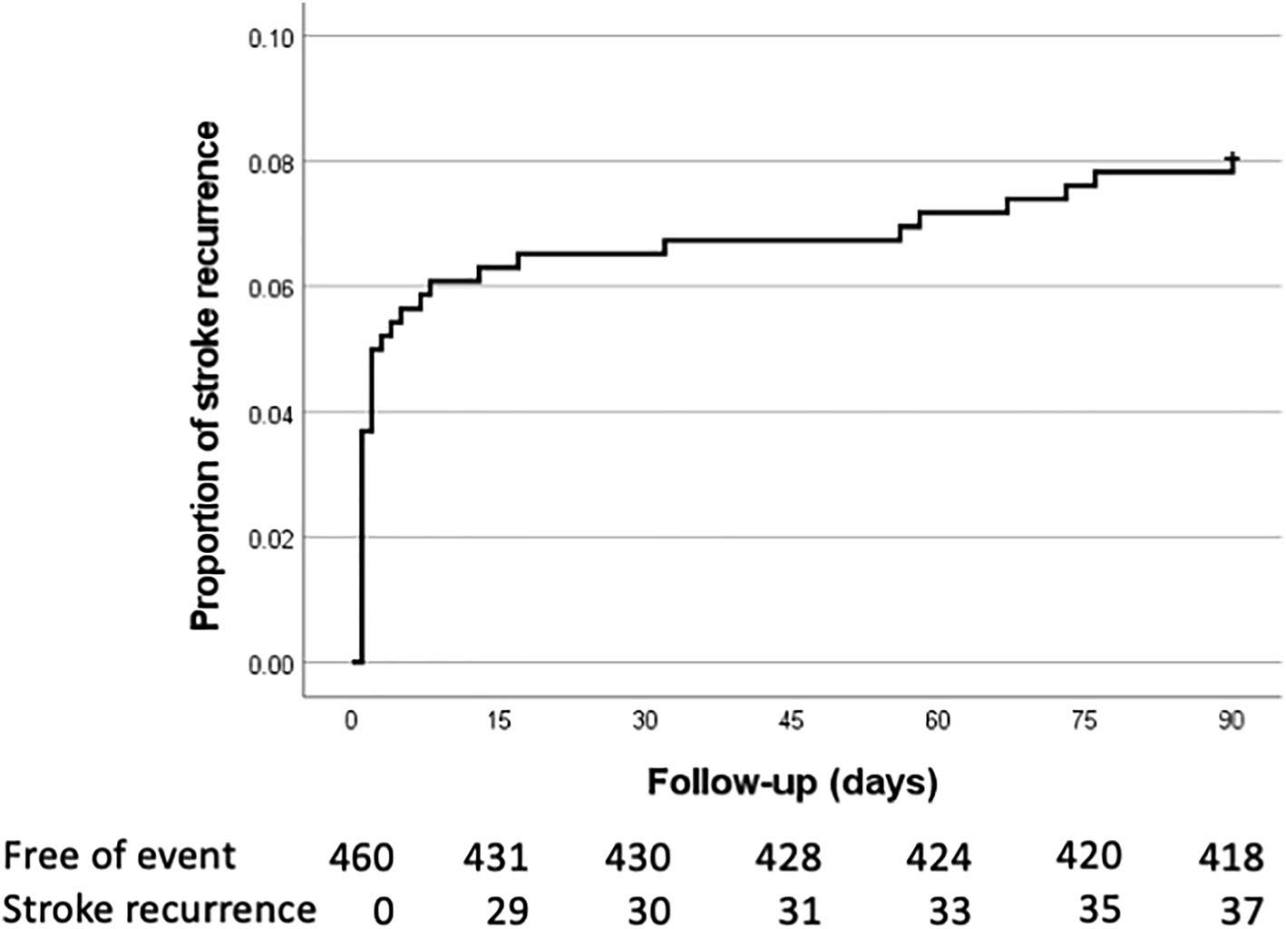
Kaplan–Meier event curves at 90 days. Proportion of patients with stroke recurrence over a period of 90 days.

As shown in Table 1, a total of 460 consecutive TIA patients upon arrival to the medical facility were included in the analysis with a mean age of 71.4 (SD 13.6) years, 221 (48.0%) patients were female. A total of 37 (8%) patients suffered SR after 90 days follow-up (Fig. 1), of whom 23 were females (62.2%).

**Table 1:** Clinical characteristics associated with stroke recurrence (SR) after 90 days follow-up.

	All N = 460	Non-SR N = 423 (92.0%)	SR N = 37 (8.0%)	*p* value^a^
Age, mean (SD)	71.4 (13.6)	71.4 (13.6)	70.4 (14.3)	0.662
Female, n (%)	221 (48.0)	198 (46.8)	23 (62.2)	0.086
Previous ischemic stroke, n (%)	32 (7.0)	25 (5.9)	7 (18.9)	*0.003*
Hypertension, n (%)	327 (71.1)	303 (71.6)	24 (64.9)	0.384
IHD, n (%)	56 (12.2)	53 (12.5)	3 (8.1)	0.43
Atrial fibrillation	112 (24.3)	101 (23.9)	11 (29.7)	0.426
Diabetes mellitus, n (%)	122 (26.5)	111 (26.2)	11 (29.7)	0.645
Smoking, n (%)	25 (5.4)	24 (5.7)	1 (2.7)	0.445
Hypercholesterolemia, n (%)	224 (48.7)	208 (49.2)	16 (43.29	0.489
Cluster TIA, n (%)	90 (19.6)	80 (18.9)	10 (27.0)	0.233
Duration				0.09
< 10’	86 (18.7)	84 (19.9)	2 (5.4)	
10’–60’	203 (44.1)	185 (43.7)	18 (48.6)	
> 60’, n (%)	171 (37.2)	154 (36.4)	17 (45.9)	
Motor impairment, n (%)	245 (53.3)	218 (51.5)	27 (73.0)	*0.012*
ABCD2 groups				
0–3	116 (25.2)	110 (26.09	6 (16.2)	0.106
4–5	252 (54.8)	233 (55.1)	19 (51.4)	
6–7	92 (20.09	80 (18.9)	12 (32.4)	
DWI abnormality, n (%)	80 (36.7)	64 (32.7)	16 (72.7)	< *0.001*
Etiology, n(%)				
LAA	94 (20.4)	81 (19.1)	13 (35.1)	*0.021*
CE	125 (27.2)	113 (29.1)	12 (32.4)	0.453
SV	130 (28.3)	123 (29.1)	7 (18.9)	0.188
Undetermined	104 (22.6)	100 (23.6)	4 (10.8)	0.099
Cholesterol total, mean (SD) mg/dL	180.2 (45.6)	180.8 (46.3)	174.5 (38.1)	0.509
LDL, mean (SD) mg/dL	111.1 (36.7)	111.4 (37.6)	107.2 (25.5)	0.639
HDL, mean (SD) mg/dL	47.4 (13.4)	47.2 (12.8)	48.9 (18.5)	0.578
Triglycerides, mean (SD) mg/dL	134.2 (86.8)	135.6 (89.0)	120.3 (61.4)	0.413
Previous statin treatment, n (%)	146 (33.7)	135 (34.0)	11 (30.6)	0.675

Plus–minus values are means ± SD. Percentages may not total 100 because of rounding.

Significant values are in italics.

^a^: student t-test.

*SR* Stroke recurrence; *IHD* Ischemic heart disease; *TIA* Transient ischemic attack; *ABCD2* Age, blood pressure, clinical features, symptom duration, and diabetes mellitus risk scores; *DWI* Diffusion-weighted imaging; *LAA* Large-artery occlusive disease; *CE* Cardioembolism; *SV* Small vessel diseases; *LDL* Low-density lipoprotein; *HDL* High-density lipoprotein; *TG* Triglycerides.

**Figure 2:**
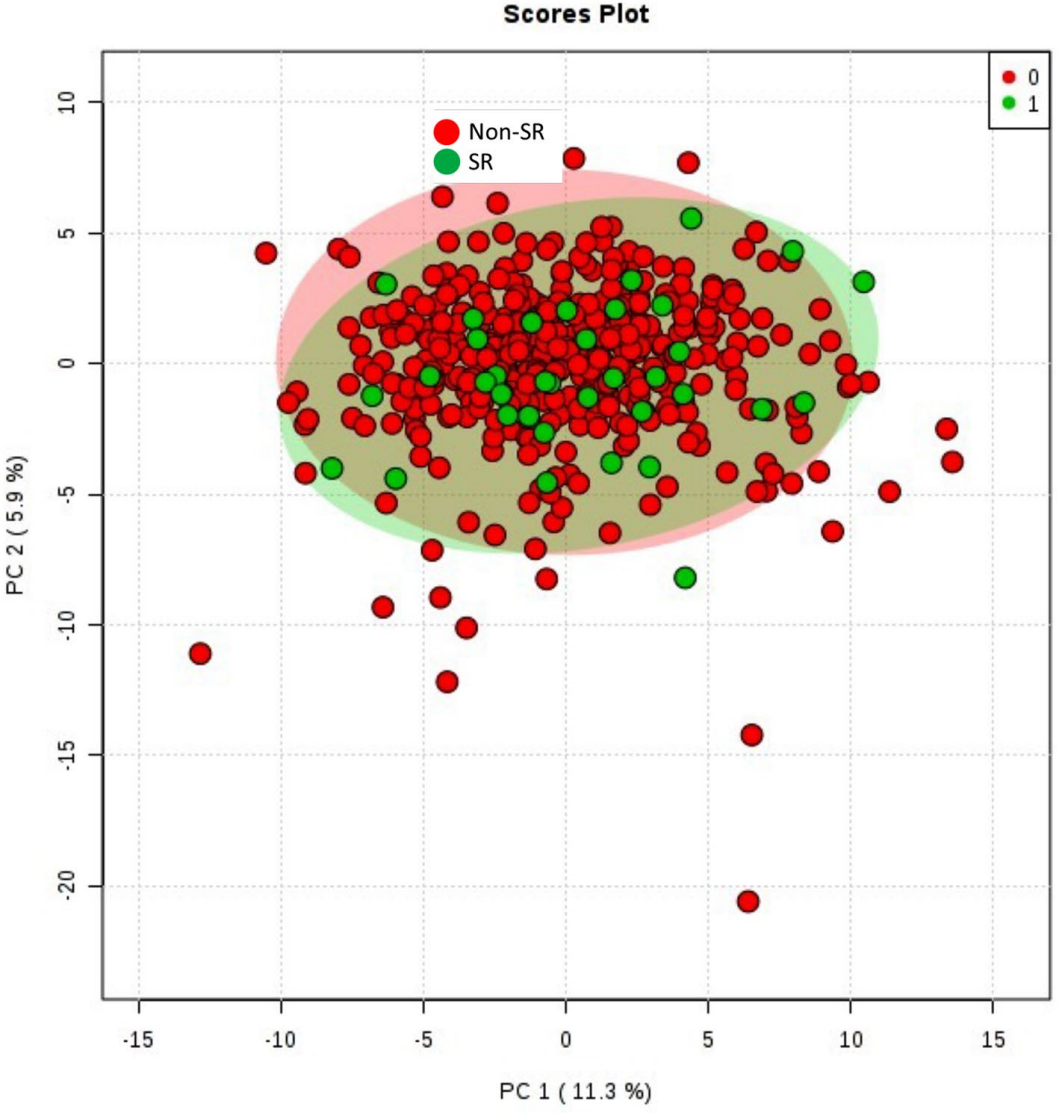
Multivariate statistics reveals little changes in plasma lipidome between stroke recurrence (SR) and non-SR patients. A. Two-dimensional Principal Component Analysis (PCA) for the different analyzed groups.

In order to have an overview of the whole lipidome, an untargeted lipidomic approach was applied. Multivariate statistics revealed small changes in the lipidome according to SR. Specifically, when unsupervised methodology (Principal Component Analyses, Fig. 2) was applied, no differences were observed between groups. Confirming that SR provokes minor changes in plasma lipidome, univariate statistics revealed that 7 of the 152 analyzed molecules (4.6%) were differentially expressed on SR from non-SR patients (Student T test, *p* < 0.05) (Table 2), all of them down-regulated. Importantly, we identified (based on exact mass, retention time and/or MS/MS spectrum) 5 triacylglycerides (TG), 1 diacylglyceride (DG), and 1 alkenyl-PE (plasmalogen) (specifically, TG(56:1), TG(63:0), TG(58:2), TG(50:5), TG(53:7, DG(38:5)) and PE(P-18:0/18:2)), arising the importance of bioenergetics molecules in SR phenomena. Interestingly, TG(56:1) and TG(63:0) were also statistically significant after false discovery range (FDR) correction.

**Table 2:** Identification of differentially expressed lipids between non-stroke recurrence (non-SR) and stroke recurrence (SR) patients.

Features	m/z	Mass	RT (min)	*p* value^a^	FDR	Regulation (SR vs. non-SR)
TG(56:1)	917.84994	916.8421	10.18423	2.05E-06	0.00031091	Down
TG(63:0)	1017.96584	1016.958	10.42379	0.00053035	0.040307	Down
TG(58:2)	960.91774	959.9099	10.18651	0.0020546	0.1041	Down
TG(50:5)^b^	842.74214	841.7343	9.549459	0.0038061	0.14463	Down
PE(P-18:0/18:2)^b^	728.58044	727.5726	7.76085	0.017047	0.51824	Down
DG(38:5)^b^	643.54474	642.5369	8.254209	0.041417	0.66839	Down
TG(53:7)	880.74514	879.7373	9.712666	0.043566	0.66839	Down

*RT, min* Retention time expressed in minutes; *FDR* False discovery range, *TG* Triglyceride, *DG* Diglyceride, *PE* phosphoethanolamine.

^a^student t-test.

^b^Confirmed by MS/MS spectra.

In parallel with the lipidomic study we identified a higher proportion of patients with sex female, previous ischemic stroke, duration of symptoms > 10 min, motor impairment, LAA and DWI abnormality in the SR group. No significant differences were observed in the standard clinical lipid profile between the two groups.

## Discussion

We observed differences in the plasma lipidomic profiling of TIA patients who suffered a subsequent SR compared to TIA non-SR patients. The lipidomic profile of patients with SR consisted of a very restrictive set of lipids made up of 5 TG, 1 DG, and 1 plasmalogen. The observed changes in these lipid classes require special attention because the metabolic pathways and cell mechanisms behind them can be crucial in the physiopathology of SR.

There are two functional categories associated with the different lipid classes identified: bioenergetics, and antioxidant protection. Thus, TG are bioenergetic compounds that compose the lipid droplets, and they are also present in neural cells^15^. DG are components of cell membranes and lipid mediators, but also precursors for biosynthesis of TG^15^. Finally, plasmalogens are structural components of cell membranes^16^ and phospholipid monolayer of LDs^17^, and they also have antioxidant properties^18^ that help to maintain lipid layer integrity.

Our results indicate a significantly low abundance of these particular lipid species in SR patients compared to non-SR subjects. The observed low abundance of particular DG and TG lipid species in plasma from SR patients points to a low accumulation/formation of cerebral LDs indicating a patient-specific response to stress conditions and suggesting a defective ischemia-associated stress response of SR patients. In another hand, the detected differential plasmalogen also requires a special attention. In human brain, phosphatidylethanolamines (PE) are quantitatively the major phospholipid^19,20^ and the predominant form is the alkenyl-PE. Plasmalogens play a key role in neural membrane properties such as membrane trafficking, cell signalling and antioxidant protection, as well as a preferential component of the phospholipid monolayer present in lipid droplets (LDs), the lipid storage organelles composed of a core of TG and sterol esters surrounded by a phospholipid monolayer and different associated proteins^21^, predominantly in glial cells and in lesser degree in neurons^22^LDs^21^. Consequently, the low abundance in plasm PE(P-18:0/18:2) from SR patients reinforces the suggested idea of alterations of LDs in SR patients, as well as an impairment in the antioxidant capacity^23^. However, more studies are needed to validate this concept, as well as the biological relevance of this particular lipid species instead of other plasmalogens. Importantly, these findings are in line with previous observations in animal models of ischemia-reperfusion^17,24^ and in ischemic stroke patients^25^, suggesting that this lipid set express a condition of impaired stress in SR patients compared to TIA non-SR patients.

Recent studies analyzing different biofluids (serum and urine) from a metabolomic approach have demonstrated, comparing stroke patients with healthy controls, the presence of specific metabolic profiles ascribed to changes in fatty acids, amino acids, choline metabolism, phospholipids, sphingolipids, and folate one-carbon cycle^25–29^. These few works collectively reveal the complexity of analyzing and discern metabolic events associated with stroke and the identification of unambiguous biomarkers. Brain ischemia occurs when there is a blockage of blood flow to the brain tissue, resulting in a decreased supply of energy to the affected area that alters membrane ionic balance, depolarizes neuronal membrane, increases intracellular Ca^2+ ^concentrations and activates calcium-dependent proteases which, ultimately, leads to the neuronal death^15,25^. Additional cell damaging mechanisms include alterations of the blood brain barrier and subsequent increase in cerebral oxidative damage and neuroinflammatory response^30^, as well as metabolic alterations affecting lipid metabolism^12^. Effectively, hypoxic stress (and other cerebral pathological states) induces a cerebral increased content of LDs predominantly in glial cells and in lesser degree in neurons^22^. This accumulation of LDs is suggested as a support for energy supply, as well as a neuroprotective mechanism against the stress-induced lipotoxicity^22^. Remarkably, diverse studies using animal models of ischemia–reperfusion demonstrated that the limited regenerative ability of the injured brain is associated with the formation of inhibitory lipids in the damaged region^17^. Consistent with this hypothesis, it seems that TIA patients who are at a higher risk of SR also exhibit a more initial pronounced ischemic insult, as indicated by a greater proportion of DWI lesions. Therefore, TIA patients with lower bioenergetic or antioxidant capacity will be more susceptible to experiencing recurrent ischemia or may have a reduced ability to recover from new ischemic episodes.

The clinical applicability of our results may be limited primarily due to the inherent complexity of the lipidomic analysis technique, which does not provide rapid results. However, the use of blood biomarkers that support stroke diagnosis and early identification of subjects with high-risk of recurrence is currently of interest^31^. Given the high prevalence of cerebrovascular disease worldwide^2^ and considering the heterogenous risk of SR among TIA patients^6,32^, the use of biomarkers related to SR could help the assessment of the individual risk of SR and management decisions^12^, especially in places without direct access to brain and/or vascular imaging.

We believe that our results, despite the limitations of the study listed below, are reproducible and representative of the clinical reality TIA patients as we included a considerable number of patients and as we identified variables previously describes with SR like motor weakness^32,33^, LAA^6,8,9,34,35^ and DWI abnormality^7,9,10^.

This work has several limitations that must be considered: (1) High-throughput lipidomic techniques have inherent handicaps such as a high variable-to-sample ratio and the high variability in the levels of metabolites and the results. Therefore, they require large sample sizes and efficient dimensionality reduction techbiques, as well as the use of validation cohorts to improve the robustness and replicability of the results. In this sense, due to the small incidence of SR sample size the statistical power of the results obtained were limited. In addition, we admit that lipidomic analysis could be influenced by many uncontrolled conditions. We highlight that only two species that pass the FDR test. Therefore, our results should be confirmed in other independent cohorts. (2) In this work we have only analyzed those lipid classes that ionize in positive mode. Therefore, the metabolites that ionize better in negative mode (such as free fatty acyls) may be underrepresented. (3) The annotation of the compounds is a well-known limitation of the untargeted lipidomic approaches. In the present work we were able to annotate 100% of the differential lipid species but 3 of 7 were not confirmed by MS/MS spectrum because they were not available in the databases. A future confirmation of these identities could change or modify the conclusions withdrawn at the biological and mechanism levels. (4) Finally, it is important to acknowledge the absence of a prior sample size calculation, although the number of events was enough to perform multivariate analysis of the clinical variables, the lipidomic analysis could be underpowered.

In conclusion, the lipidomic profiles of TIA subjects with non-SR and SR were different, with minor but significant changes. The observed changes in lipid patterns, especially PE(P-18:0/18:2), suggest pathophysiological mechanisms associated with LDs metabolism and antioxidant protection that is translated to plasma level as consequence of a more intensive or high-risk ischemic condition related to early SR. The determination of these differential metabolites which are related to bionerergetics pathways and oxidative stress could improve the assessment of individual risk of SR and management decisions. In addition, our findings encourage the investigation of new potential pharmacological interventions.

## Material and methods

### Design and study population

We developed a registry-based cohort study following the Strengthening the Reporting of Observational Studies in Epidemiology (STROBE) statement^36^. We included consecutive TIA patients attended by a stroke neurologist working at the emergency department of a hospital during the first 24 h after the onset of symptoms from January 2006 to January 2015. TIA was defined according to the World Health Organization criteria as a reversible episode of neurological deficit of ischemic origin that was fully resolved within 24 h^37^. In all cases, the nature of the transient symptoms was evaluated for the final diagnosis of TIA after neuroimaging assessment. If patients were fully recovered from symptoms on arrival to the hospital, the precise neurologic symptoms and its duration were determined by interviewing the patients, family members or other caregivers. A structured questionnaire was used to record criteria accordingly to the Reduction of Atherothrombosis for Continued Health Registry (REACH)^38^ the following variables: age, sex, vascular risk factors (hypertension, diabetes mellitus, hyperlipidemia, current smoking habit), and previous vascular disease including documented coronary artery disease and peripheral artery disease^35^. In patients who underwent magnetic resonance imaging (MRI) a trained radiologist with access to clinical information but blinded to patient outcomes analyzed the presence of diffusion-weighted imaging (DWI) abnormalities. Peripheral venous samples were obtained within the first 24 h from symptom onset.

### Outcomes and follow-up

The primary outcome was the occurrence of SR. It was defined as a new symptomatic neurologic deficit that was not attributable to a nonischemic cause accompanied by neuroimaging evidence of a new brain infarction. Structured clinical visits were performed by a stroke physician during the follow-up period at 90 days. All patient events, death records, electronic medical records, hospital admissions records were reviewed, and in needed cases the primary care physician was consulted^35^.

### Classification of stroke subtypes

Patients were classified etiologically based on the TOAST classification of stroke subtypes (REF; SSS-TOAST, an evidence-based causative classification system for ischemic stroke)^39^ at the 90 days follow-up visit after the evaluation of all available test results by a stroke neurologist. The identified etiologies were large-artery occlusive disease (LAA), small-vessel disease, cardioembolic, uncommon or undetermined causes. Patients were classified as LAA if they exhibited a symptomatic, moderate to severe, intracranial or extracranial stenosis^7^. We applied the small artery disease classification to patients with no evidence of LAA or cardioembolic TIA who reported classic lacunar syndrome (pure motor, pure sensory, and sensorimotor syndrome involving at least 2 out of 3 specific body parts -face, arm, and leg-) and ataxic hemiparesis or dysarthria-clumsy hand syndrome^40^.

### Lipidomics approach

Untargeted lipidomic analyses was performed using an Agilent 1290 LC system coupled to an electrospray-ionization quadruple time of flight mass spectrometer (Q-TOF, 6520 instrument, Agilent Technologies, Barcelona, Spain).

Plasma lipid species were extracted using a MTBE based methodology as described previously^41^. For protein precipitation, five μl of Mili Q water and 20 μl of methanol were added to 10 μl of a plasma sample and shaken for 2 min, and then 50μL of methyl tert-butyl ether (MTBE), containing internal lipid standards (Table S1) were added. Samples were immersed in a water bath (ATU Ultrasonidos, Valencia, Spain with an ultrasound frequency and power of 40 kHz and 100 W, respectively, at 10 °C for 30 min. Then, 75 μL of Mili Q water was added to the mixture, and the organic phase was separated by centrifugation (1400 g) at 10 °C for 10 min. The upper phase, containing all the extracted lipid species, was collected, and subjected to analyses. A pool of all lipid extracts was prepared and used as QC as previously described^42^.

Internal isotopically labeled lipid standards for each class were used for signal normalization^43^ Ten μl of lipid extract was applied onto 1.8 μm particle 100 × 2.1 mm id Waters Acquity HSS T3 column (Waters, Milford, MA, USA) heated at 55 °C. The flow rate was 400 μl/min with solvent A composed of 10 mM ammonium acetate in acetonitrile–water (40:60, v/v) and solvent B composed of 10 mM ammonium acetate in acetonitrile-isopropanol (10:90, v/v). The gradient started at 40% B and reached 100% B in 10 min and held for 2 min. Finally, the system was switched back to 40% B and equilibrated for 3 min, as previously described^44^.

Data were collected in positive electrospray mode TOF operated in full-scan mode at 50–3000 m/z in an extended dynamic range (2 GHz), using N_2_ as the nebulizer gas (5 L/min, 350 °C). The capillary voltage was 3500 V with a scan rate of 1 scan/s. The ESI source used a separate nebulizer for the continuous, low-level (10 L/min) introduction of reference mass compounds 121.050873 and 922.009798, used for continuous, online mass calibration. Mass Hunter Data Analysis Software (Agilent Technologies, Barcelona, Spain) was used to collect the results, and Mass Hunter Qualitative Analysis Software (Agilent Technologies, Barcelona, Spain) to obtain the molecular features of the samples, as described^12^. We selected features with a minimum of 2 ions (adducts) to ensure that the feature corresponds to a specific metabolite. MassHunter Mass Profiler Professional Software (Agilent Technologies, Barcelona, Spain) was used to select, align, and filter molecular features. Multiple charge states were considered. Compounds from different samples were aligned using a retention time window of 0.1% ± 0.25 min and a mass window of 30.0 ppm ± 2.0 mDa. We selected only those features that are present in 100% of QC and had a maximum RSD among QC of 20%. Samples were normalized using a LOESS-based approach^45^. After outlier analyses 452 individuals (415 non-SR vs. 37 SR) were selected to apply both multivariate and univariate statistics. Baseline correction, peak picking and peak alignment were performed on acquired data. After quality control assessment, filtering (we chose only those features that are present in 100% of quality controls (QC) and had a maximum robust standard deviation (RSD) among QC of 20%) and correcting the signal, 152 features remained (supplementary dataset), which were used for multivariate and univariate statistical analysis. Identities were confirmed based on exact mass, retention time, isotopic distribution, and MS/MS spectrum using public databases such as Metlin^46^, HMDB^47^, and LipidMatch^48^. Because we applied a semiquantitative approach, the results are offered as relative abundance (MS counts).

### Statistical analysis

We compared the baseline characteristics, etiology, presence of acute lesions in DWI, between non-SR and SR patients. The quantitative variables were compared using either the student’s T-test or the Mann–Whitney U test. The qualitative variables were compared using the chi-squared test or Fisher’s exact test when the expected frequency was less than 5. The statistical analysis of the data was carried out using the SPSS statistical package, version 24.0. (SPSS, Chicago, IL, USA). Statistical significance was considered when *p* < 0.05. In addition, we find differences in the lipidomic profile in plasma samples between patients with and without SR. For this purpose metaboanalyst platform^49^ was used to perform univariate and multivariate statistics (PCA) of the extracted features.

### Standard protocol approvals, registrations, and patient consents

The local ethics committee approved the TIA registry. Written informed consent was obtained from all participants or their designated representative^35^.

### Ethical approval

The study was conducted according to the guidelines of the Declaration of Helsinki and approved by the Institutional Review Board (or Ethics Committee) of Hospital del Mar–Parc Sanitari Mar (protocol code 2008/3084/I).

### Informed consent

Informed consent was obtained from all subjects involved in the study.

### Supplementary Information

Supplementary Information.

Supplementary Table S1.

## Data Availability

Requests for access to the data reported in this article will be considered by the corresponding author.
