# Supplementary material for: Lipidomic signature of stroke recurrence after transient ischemic attack
Source: Sci Rep. 2023 Aug 22;13:13706. doi: 10.1038/s41598-023-40838-7 (PMC10444771; doi:10.1038/s41598-023-40838-7)
Supplement: Supplementary file 2 — Supplementary Table S1. [file 41598_2023_40838_MOESM2_ESM.docx]

**Table S1**. Class representative and extraction internal standards added to the samples in untargeted lipidomics analysis.

| COMPOUND | SOURCE | IDENTIFIER |
| --- | --- | --- |
| 1,3(d5)-dihexadecanoyl-glycerol | Avanti Polar Lipids | 110537 |
| 1,3(d5)-dihexadecanoyl-2-octadecanoyl-glycerol | Avanti Polar Lipids | 110543 |
| 1-hexadecanoyl(d31)-2-(9Z-octadecenoyl)-sn-glycero-3-phosphate | Avanti Polar Lipids | 110920 |
| 1-hexadecanoyl(d31)-2-(9Z-octadecenoyl)-sn-glycero-3-phosphocholine | Avanti Polar Lipids | 110918 |
| 1-hexadecanoyl(d31)-2-(9Z-octadecenoyl)-sn-glycero-3-phosphoethanolamine | Avanti Polar Lipids | 110921 |
| 1-hexadecanoyl-2-(9Z-octadecenoyl)-sn-glycero-3-phospho-(1'-rac-glycerol-1',1',2',3',3'-d5) | Avanti Polar Lipids | 110899 |
| 1-hexadecanoyl(d31)-2-(9Z-octadecenoyl)-sn-glycero-3-phospho-myo-inositol | Avanti Polar Lipids | 110923 |
| 1-hexadecanoyl(d31)-2-(9Z-octadecenoyl)-sn-glycero-3-[phospho-L-serine] | Avanti Polar Lipids | 110922 |
| 26:0-d4 Lyso PC | Avanti Polar Lipids | 860389 |
| 18:1 Chol (D7) ester | Avanti Polar Lipids | 111015 |
| cholest-5-en-3ß-ol (d7) | Avanti Polar Lipids | LM-4100 |
| D-erythro-sphingosine-d7 | Avanti Polar Lipids | 860657 |
| D-erythro-sphingosine-d7-1-phosphate | Avanti Polar Lipids | 860659 |
| N-palmitoyl-d31-D-erythro-sphingosine | Avanti Polar Lipids | 868516 |
| N-palmitoyl-d31-D-erythro-sphingosylphosphorylcholine | Avanti Polar Lipids | 868584 |
| Octadecanoic acid-2,2-d2 | Sigma Aldrich | 19905-58-9 |
